# Dr. Zira Hazard Potter

**Published:** 1913-05

**Authors:** 


Dr. Zira Hazard Potter, Geneva, 1857, formerly on the faculty of Cornell University, died April 1, aged 76, at Bethlehem, Pa.

